# Rapid response to a coronavirus disease 2019 (COVID-19) outbreak in a psychiatry hospital—Kanazawa City, Japan, March to April 2020

**DOI:** 10.1017/ash.2021.255

**Published:** 2022-04-12

**Authors:** Hirofumi Kato, Kikuno Seki, Yoshiki Maeda, Yoko Noda, Yoshitsugu Iinuma, Masami Kitaoka, Keisuke Kiso, Rie Koshida, Hitomi Kurosu, Takuya Yamagishi, Tomoe Shimada, Motoi Suzuki, Tomimasa Sunagawa

**Affiliations:** 1 Department of Virology, National Institute of Infectious Diseases, Tokyo, Japan; 2 Infection Control Team, Okabe Hospital, Ishikawa, Japan; 3 Infection Control Team, Kanazawa Medical University, Ishikawa, Japan; 4 Department of Infectious Diseases, Kanazawa Medical University, Ishikawa, Japan; 5 Kanazawa City Public Health and Welfare Bureau, Ishikawa, Japan; 6 Antimicrobial Resistance Research Center, National Institute of Infectious Diseases, Tokyo, Japan; 7 Center for Field Epidemiology Intelligence, Research, and Professional Development, National Institute of Infectious Diseases, Tokyo, Japan; 8 Center for Surveillance, Immunization, and Epidemiologic Research, National Institute of Infectious Diseases, Tokyo, Japan

## Abstract

A coronavirus disease 2019 (COVID-19) outbreak in a psychiatry hospital revealed specific challenges in its response such as difficulty in isolation, transfer, and identification of close contacts, suboptimal infection control practices, and shortage of personal protective equipment, which were overcome by support from the public health center and a neighboring university hospital.

To address coronavirus disease 2019 (COVID-19) outbreaks, the Japanese government has applied a cluster-based prevention approach that features intensive contact tracing and targeted testing strategy.^1^ Among 61 clusters occurring in Japan through April 2020, 18 (30%) healthcare facilities and 10 (16%) other care facilities, such as nursing homes and daycare centers, were identified. The fatalities in these facilities,^2^ where most patients and residents were elderly, affected many communities.^3^ Among healthcare facilities, psychiatric facilities required special management due to their unique characteristics.^4–8^ In March and April 2020, Kanazawa City Public Health and Welfare Bureau (KCPHWB), National Institute of Infectious Diseases, and Kanazawa Medical University conducted an outbreak investigation in a psychiatry hospital and supported implementation of infection prevention and control (IPC) measures. Here, we report descriptive epidemiology and challenges of the outbreak response for this unique population.

## Materials and methods

The psychiatry hospital has 6 wards with 294 licensed beds, ∼85% of which are in multibed room (3–6 patients). At the time of the study, 234 healthcare workers (HCWs) worked full time or part time in these wards. The hospital provides several functions for outpatient care and inpatient treatments including acute- and long-term care for psychiatric emergencies. A chief nurse who had received lectures and training from external IPC specialists was in charge of IPC.

In accordance with the case definition at that time,^9^ a confirmed COVID-19 case was defined as an HCW and patient at the psychiatry hospital between March 26 and April 30, 2020, with a positive nasopharyngeal specimen for SARS-CoV-2 by polymerase chain reaction (PCR) assay. Close contacts identified through the investigation, and HCWs and inpatients who developed symptoms during the aforementioned period underwent PCR testing. For HCWs, a chief nurse collected samples outside in a drive-through setting with appropriate personal protective equipment (ie, medical mask, face shield, gown, and gloves). For inpatients, 1 or 2 additional HCWs wearing PPE helped to hold the head and/or body of the patient during sample collection.

After detection of the index case, KCPHWB conducted active case finding including testing and interviews with cases and their close contacts to record demographic factors and symptoms within the 2 weeks before symptom onset. Clinical information of confirmed cases was retrospectively collected from medical records. This investigation was conducted under the law as part of the public health response, and no consent was obtained from the individuals.

## Results

**Fig. 1.**
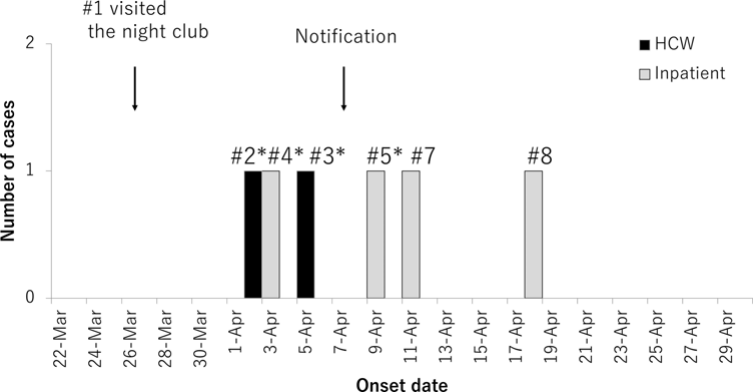
Timeline of the COVID-19 outbreak in the psychiatry hospital, March 22 through April 29, 2020 (n = 6). Confirmed cases were excluded when the onset dates were unknown. Black bars indicate inpatients identified as confirmed cases; hatched bars indicate healthcare workers (HCWs) identified as confirmed cases. Arrows indicate the dates that case 1 visited the night club and Kanazawa City Public Health and Welfare Bureau was notified of the outbreak. Asterisks indicate contact with the index case.

On March 26, 2020, the index case visited a night club (where a COVID-19 outbreak was later announced to the public) and developed respiratory symptoms. On April 3, he developed other respiratory symptoms. He continued to work but finally tested positive on April 8 (Fig. 1). An active case investigation found that 8 cases met the case definition (Table 1). The median age was 57.5 years (range, 39–85); 5 cases were male (63%); and 2 cases were fatal (case fatality rate, 25%). All cases presented typical symptoms, such as fever and/or respiratory symptoms, but 2 had neuroleptic malignant syndrome or seasonal allergy. Among these 8 cases, 3 (38%) were medical doctors and 5 (63%) were inpatients. Of the inpatients, 4 were hospitalized in the same long-term care unit (M-1), and 1 patient was in a different unit (E-2) that provided acute care. Due to a shortage of beds for COVID-19 cases with psychiatric disease in designated hospitals, 4 cases were transferred only after they became severely ill.

**Table 1.** Characteristics of the COVID-19 Cases in an Outbreak at a Psychiatry Hospital in Japan, March 26 through April 30, 2020 (n = 8)

No.	Age/Sex	Case definition	Onset date	Notification date	Epidemiological linkage	Affiliation	Ward
1	30s, M	Confirmed	N/A	April 7	Visited the night club	Doctor	N/A
2	40s, M	Confirmed	April 2	April 7	Contact with case 1	Doctor	N/A
3	30s, M	Confirmed	April 5	April 7	Contact with case 1	Doctor	N/A
4	60s, F	Confirmed	April 3	April 7	Contact with case 1	Inpatient	M-1
5	80s, M	Confirmed	April 9	April 10	Contact with case 1	Inpatient	M-1
6	60s, F	Confirmed	N/A	April 12	Unknown	Inpatient	E-2
7	50s, M	Confirmed	April 11	April 12	Unknown	Inpatient	M-1
8	60s, F	Confirmed	April 18	April 20	Moved to the room case 4 had stayed	Inpatient	M-1

Note. M, male; F, female; N/A, not applicable; M-1, 1st floor in main building; E-2, 2nd floor in east building.

The index case had close contact with cases 2 and 3 in their recreation room, with case 4 in group therapy, and with case 5 in the examination room. Cases 6 and 7 did not have clear contact with the confirmed cases, and the transmission route was not identified. Case 8 moved into the room just occupied by case 4 and had no clear contact with the confirmed cases.

All HCWs and inpatients in M-1 and E-2 wards were considered close contacts because inpatients moved freely within wards, ate in the group dining hall, and participated in group therapy. In total, 169 HCWs and inpatients received a PCR test.

In addition to prohibiting visitors from outside, the hospital enhanced control measures including (1) closing outpatient clinic and care services, (2) restricting staffing to essential HCWs only and prohibiting symptomatic HCWs from working, and (3) discontinuation of group dining and group therapy. Instead, the hospital provided other rehabilitation programs that the patient did in their own rooms (eg, paper work). To strengthen IPC, the following measures were undertaken: (1) immediate isolation of cases and symptomatic patients in a single room or fixed area with the doors closed, (2) cohorting of close contacts with appropriate preventive measures, (3) strengthening of daily health monitoring of inpatients, (4) recommending that HCWs avoid closed spaces, crowded places, and close-contact settings, (5) ensuring universal masking among HCWs, and (6) supplying adequate PPEs. The university IPC unit supported strengthening IPC at the hospital and conducted remote online consultation.

## Discussion

In most closed or semiclosed facilities such as psychiatry hospitals,^8^ severe acute respiratory coronavirus virus 2 (SARS-CoV-2) is frequently introduced from outside, mostly by HCWs, suggesting that preventing HCW infection outside the healthcare setting is extremely important to reduce outbreaks within psychiatry hospitals. Difficulty in obtaining information from patients, enclosed and crowded wards, unrecognized interaction among patients, and many group activities resulted in the conclusion that all HCWs and inpatients were close contacts in the affected wards. Similar situations may exist in facilities treating the disabled and nursing homes where contact details are usually unavailable.

Strengthening IPC measures was a real challenge for the following reasons: (1) insufficient cognitive abilities of inpatients to take appropriate self-infection control measures, (2) potential communication barrier between inpatients and HCWs wearing PPE, which may influence the patient’s psychiatric condition, (3) unfamiliarity with basic IPC measures among HCWs, and (4) shortages of PPE. Repeated on-site and remote training for HCWs by IPC specialists in a neighboring hospital helped to improve the IPC levels relatively quickly and contributed to the termination of the outbreak.

Another challenge was the limited number of substitute HCWs with psychiatric nursing skills and beds in neighboring, designated hospitals with the capacity to treat patients with both psychiatric disease and COVID-19. Furthermore, patients who could not be transferred to designated hospitals caused a heavy workload in the hospital. A local network of IPC and psychiatric care specialists (eg, infection control nurses or doctors in a university hospital) greatly helped in addressing these problems.

Despite the possibility of unrecognized transmission routes, only 1 inpatient case was identified. The following measures enabled us to contain the outbreak earlier than we expected: rapid isolation of the cases, expansion of close contact tracing, comprehensive testing of HCWs, strengthening of IPC measures, supplying adequate PPE, and rapid information sharing among stakeholders. They gradually resumed their services 1 month later. Spillover from the hospital was not observed nor did additional outbreaks occur at the hospital thereafter.

This study had several limitations. Misclassification may have occurred because of the intermediate sensitivity of PCR testing.^10^ Information bias about contacts may have occurred due to the retrospective nature of the investigation.

In summary, during a COVID-19 outbreak in a psychiatry hospital, a rapid response team led by the local public health authority and networking among various external partners may help overcome difficulties in contact tracing and safe specimen collection, shortages of beds and HCWs specializing in psychiatric care, and HCW unfamiliarity with IPC measures. Our comprehensive measures will be effective in preventing further outbreaks of COVID-19.
